# Hypomethylation-driven AKT Serine/Threonine Kinase 3 promotes testicular germ cell tumors proliferation and negatively correlates to immune infiltration

**DOI:** 10.1080/21655979.2021.2002621

**Published:** 2021-12-09

**Authors:** Yang Luo, Qianyin Zhou, Fang Zhu, Liqing Fan, Hao Bo, Xingming Wang

**Affiliations:** a Department of Obstetrics and Gynecology, Center for Reproductive Medicine, Key Laboratory for Major Obstetric Diseases of Guangdong Province, the Third Affiliated Hospital of Guangzhou Medical University, Guangzhou, Guangdong, China; b Key Laboratory for Reproductive Medicine of Guangdong Province, the Third Affiliated Hospital of Guangzhou Medical University, Guangzhou, Guangdong, China; c NHC Key Laboratory of Human Stem Cell and Reproductive Engineering, Institute of Reproductive and Stem Cell Engineering, School of Basic Medical Science, Central South University, Changsha, Hunan, China; d Clinical Research Center for Reproduction and Genetics in Hunan Province, Reproductive and Genetic Hospital of CITIC-Xiangya, Changsha, Hunan, China; e Department of Nuclear Medicine (Pet Center), Xiangya Hospital, Central South University, Changsha, Hunan, China

**Keywords:** TGCT, AKT3, methylation, proliferation, immunity

## Abstract

AKT Serine/Threonine Kinase 3 (AKT3) has been reported to play an important role in different tumors. However, its clinical value, biological function, and molecular mechanism in testicular germ cell tumors (TGCT) remains unclear. In the current study, we applied the Gene Set Cancer Analysis (GSCA), UCSC XENA, Gene Expression Omnibus (GEO), the Human Protein Atlas (HPA), LinkedOmics, DiseaseMeth version 2.0, TISIDB, and other databases for TGCT data mining. Then, we investigated AKT3’s mechanism of action and clinical survival significance via bioinformatics followed by *in vitro* experiments. We found that AKT3 was upregulated and had frequent copy number amplifications in TGCT, which were associated with poor survival outcomes of patients. On the other hand, mutations that led to AKT3 loss-of-function were correlated to a better prognosis in patients. Moreover, AKT3 silencing significantly inhibited the proliferation, DNA synthesis and colony formation of NCCIT cells (a TGCT cell line). AKT3 might participate in TGCT progression through multiple signaling pathways, such as ErbB, oxidative phosphorylation, and affecting tumor immune infiltration. Also, the upregulation of AKT3 mRNA expression might be driven by the hypomethylation of its promoter region. Overall, AKT3 is a potential TGCT oncogene and can be further used as a therapeutic target.

## Introduction

Testicular germ cell tumors (TGCT) are malignant solid tumors frequently occurring in men from 15 to 40 years [1]. Histologically, TGCT have two major subtypes: seminomas (SE) and non-seminomas (NSE). It is believed that NSE originates from earlier gonadal stem cells and SE from later gonadal stem cells [2]. Besides, NSE is more likely to metastasize due to its lower sensitivity to chemotherapy and radiotherapy, resulting in more adverse outcomes [3]. Currently, TGCTs are managed mainly by surgery, radiotherapy, and cisplatin-based chemotherapy, which can contribute to an overall cure rate of up to ~80% [4]. Unfortunately, 20% of patients respond incompletely to such treatments, have a risk of recurrence and 15% will have a refractory disease [5]. Therefore, understanding TGCT’s occurrence and development, and the mechanisms behind their metastasis and recurrence in a comprehensive fashion are required to develop more effective therapeutic strategies.

AKT3, is an AKT subtype, is composed of two different splice variants: AKT3/+S472 and AKT3/-S472. Generally, AKT3/-S472 leads to a protein lacking a phosphorylation site at S472 [6], and AKT phosphorylated at this spot can negatively regulate cell apoptosis [7]. Additionally, the crucial functional roles of AKT3 in several tumors have been well investigated. For instance, AKT3 can be regulated by miR-582-5p to promote tumor cell proliferation in gastric cancer [8]. Besides, the activation of the miR-665/AKT3 signaling pathway can enhance the proliferation and metastasis of ovarian tumor cells [9]. Furthermore, increasing evidence indicates that AKT3 can be an oncogene in many cancers, including osteosarcoma [10], colorectal cancer [11], prostate cancer [12], and breast cancer [13]. In TGCT, the TR4/AKT3 signaling was identified as a potential promotor of tumor metastasis [14]. Nevertheless, the clinical significance and specific role of AKT3 in TGCTs remains unclear. Thus, we hypothesized that AKT3 would be highly expressed in TGCT patients, and this high expression would be driven by DNA copy number amplification and hypomethylation. Additionally, AKT3 would participate in the proliferation, colony formation, immune infiltration, and drug sensitivity of TGCT through different signaling pathways. Therefore, in the current study, we evaluated AKT3’s possible roles and mechanisms in TGCT via data mining and *in vitro* experimental verification to provide new ideas for future treatment strategies.

## Materials and methods

### Genomic variation and expression analysis of AKT3 in TGCT

AKT3 gene copy number variations (CNVs) and the corresponding associations with AKT3 mRNA expression and survival outcome in TGCT patients were analyzed using the data available on the Gene Set Cancer Analysis (GSCA) online software (http://bioinfo.life.hust.edu.cn/GSCA/#/) and based on the TGCT dataset from The Cancer Genome Atlas (TCGA) project with default parameters [15]. Then, AKT3 mRNA differential expression data in TGCT were retrieved from the Gene Expression Omnibus (GEO) database (accession number: GSE3218) [16]. The detailed information on TCGA (TCGA-TGCT) and GEO (GSE3218) datasets are as Table 1. Correlation analyses between AKT3 mRNA expression and survival in TGCT patients were implemented via the Kaplan-Meier Plotter [17] online tool (http://kmplot.com/analysis/index.php?p=background) and based on the TCGA-TGCT dataset. AKT3 protein levels data in normal testis and TGCT were retrieved from the Human Protein Atlas (HPA) [18] database (https://www.proteinatlas.org/). The proportion of positive cells per unit area is used to determine the difference of AKT3 expression among groups. The mutation frequency of AKT3 in TGCT patients and its associations with clinicopathological characteristics were analyzed by the cBioPortal [19] online tool (https://www.cbioportal.org/).

**Table 1.** The detailed information on TCGA-TGCT and GSE3218 datasets

Group	GSE3218	TCG-TGCT
Normal	6	0
Seminoma	13	68
Embryonal carcinoma	15	26
Yolk sac tumor	10	4
Teratoma	16	11
Choriocarcinoma	2	0
Mixed TGCTs	44	30
Total	106	139

### Cell culture and siRNA-silencing

The human TGCT cell line, NCCIT (NSE cell line), was the most widely used and most common TGCT cell line and NCCIT cells have stronger proliferation and clone formation capabilities. We obtained the NCCIT cell line from proffessor Suren Chen. First, cells were grown in RPMI-1640 medium (Gibco) containing 10% fetal bovine serum (FBS, Gibco), 100 U/mL penicillin, and 100 μg/mL streptomycin (Gibco) in a constant temperature and humidity incubator at 37 °C and 5% CO_2_. Cells growing logarithmically were collected and transferred to a 6-well plate (5.0 × 10^5^ cells/well). Then they were processed by small interfering RNA (siRNA) transfection until the cell density reached about 70%. According to the manufacturer’s instructions, AKT3 siRNA (siRNA1, siRNA2) and scrambled siRNA were transfected into cells using the Lipofectamine 3000 transfection reagent to generate experimental and control groups, respectively. After 48 h of transfection, cells were collected for subsequent experiments. All siRNAs were designed and synthesized by Guangzhou RiboBio.

### RNA extraction and qRT-PCR

Total RNA of the NCCIT cells was extracted by TRIzol (Invitrogen). One μg of RNA was used as a template, and the first-strand complementary DNA (cDNA) synthesis was completed using the Transcriptor First Strand cDNA Synthesis Kit (Roche). Next, the cDNA was processed for qRT-PCR using the LightCycler 480 PCR instrument (Roche), according to the LightCycler 480 SYBR Green I Master (Roche) experimental instructions. The AKT3 mRNA expression level, relative to β-actin, was calculated by the 2^−ΔΔCT^ method. The primers were designed and synthesized by Shanghai Sangon as follows: AKT3 forward: 5’ – ACCGCACACGTTTCTATGGT-3’, reverse: 5’ – CCCTCCACCAAGGCGTTTAT-3’; β-actin forward: 5’ – TCACCAACTGGGACGACATG-3’, reverse: 5’ – GTCACCGGAGTCCATCACGAT – 3’.

### MTT

After transfection, NCCIT cells were inoculated into a 96-well plate (1 × 10^3^ cells/well – 100 μL) and transferred to a constant temperature and humidity incubator. At 6 h, 1, 2, 3, 4, and 5 d, 10 μL of MTT solution was added into each well, then cultured for additional 4 hours. The absorbance at 450 nm was measured by enzyme-linked immunosorbent assay to determine the cell proliferation status in each well.

### EdU

After 72 h of transfection, NCCIT cells were transferred to a medium containing the EdU reagent for 2 h. Then, the medium was discarded and replaced by 4% paraformaldehyde to fix the cells at room temperature for 20 min, followed by 2 mg/mL glycine solution to neutralize and 0.5% Triton X-100. Next, the plate was washed twice with PBS. The 1× Apollo 643 staining solution was prepared according to instructions, then added into wells for 30 min at normal temperature, and protected from light. The staining solution was replaced by 0.5% Triton X-100, and the plate was washed on a shaker (2 to 3 times, 10 min each). The permeate was discarded and the plate was washed twice with PBS. Finally, 1× DAPI reaction solution was added, and cells were incubated for 30 min at room temperature in the dark. The reaction solution was removed, and the plate was washed 3 times with PBS. Images were captured by Acumen X3.

### Colony formation assay

First, cells were harvested 36 h after transfection and seeded into a 6-well plate (5 × 10^2^ cells/well). Three repetitions were performed for each group. For cell culture, a constant temperature incubator was used and the complete medium was replaced once during the process. After 2 weeks, the medium was discarded and the plate was washed twice with PBS. Subsequently, 4% paraformaldehyde was used to fix cells (30 min), followed by crystal violet staining solution (1 mL) at room temperature (15 min). Finally, images were photographed.

### Gene co-expression and pathway enrichment analyses

LinkedOmics online tool contains multi-omics data and clinical data for cancer patients from TCGA project. We can use this tool to analyze the co-expressed genes and regulatory networks of target genes. Moreover, this tool is also directly linked to the WebGestalt database, which can facilitate the enrichment analysis and visualization data. For AKT3 gene co-expression analysis, the LinkedOmics [20] online tool was applied based on the TCGA-TGCT cohort data, along with the Spearman correlation test to identify the top 50 most positively or negatively genes correlated with AKT3 (visualized by a heat map). For biological analysis, Gene Set Enrichment Analysis (GSEA) was performed using the LinkedOmics online tool based on GO and KEGG databases. Gene co-expression and pathway enrichment analyses were implemented with default parameters. A false discovery rate (FDR) of less than 0.05 is determined to be statistically significant.

### AKT3 methylation analysis

The CpG islands around the AKT3 gene promoter were profiled by the UCSC Genome online tool (https://genome.ucsc.edu/). The DNA methylation data of the AKT3 gene was retrieved from the DiseaseMeth version 2.0 database (http://bio-bigdata.hrbmu.edu.cn/diseasemeth/). The correlation between AKT3 mRNA and methylation levels was retrieved from the UCSC XENA database [21] based on the TCGA-TGCT cohort.

### Correlation of AKT3 mRNA with immune cells, molecules, infiltration, and drug sensitivity

Correlation analyses for AKT3 mRNA were performed based on the TCGA-TGCT cohort data using default parameters. The TISIDB [22] database (http://cis.hku.hk/TISIDB/index.php) was applied to characterize AKT3 mRNA associations with immune cells and molecules, and the Gene Set Cancer Analysis (GSCA) online software was used to identify its associations with immune infiltration and drug sensitivity.

### Statistical analyses

Differences between two groups were analyzed by the Student’s t-test, and among more than two groups by analysis of variance (ANOVA). The survival significance was determined by a *p* < 0.05 in the log-rank test. Histograms and Broken Line Charts construction and statistical analyses were performed using the GraphPad Prism v.5 software.

## Results

In this study, we hypothesized that AKT3 would be abnormally expressed in TGCT and regulated by copy number variation and DNA methylation. Thus, it would participate in the proliferation, colony formation, immune infiltration, drug sensitivity, and other TGCT processes. Therefore, we used GSCA, UCSC XENA, GEO, HPA, and DiseaseMeth for data mining. We found that the high AKT3 expression can be related to its copy number amplification and DNA hypomethylation. Additionally, our *in vitro* experiments confirmed that AKT3 can promote the proliferation and colony formation of TGCT cells. LinkedOmics and TISIDB database analyses revealed that AKT3 might be related to the activation of multiple oncogenic signaling pathways and immune infiltration in TGCT. These results suggested that AKT3 can be a potential target for TGCT treatments.

### High AKT3 expression is related to poor outcomes of TGCT patients

**Figure 1.**
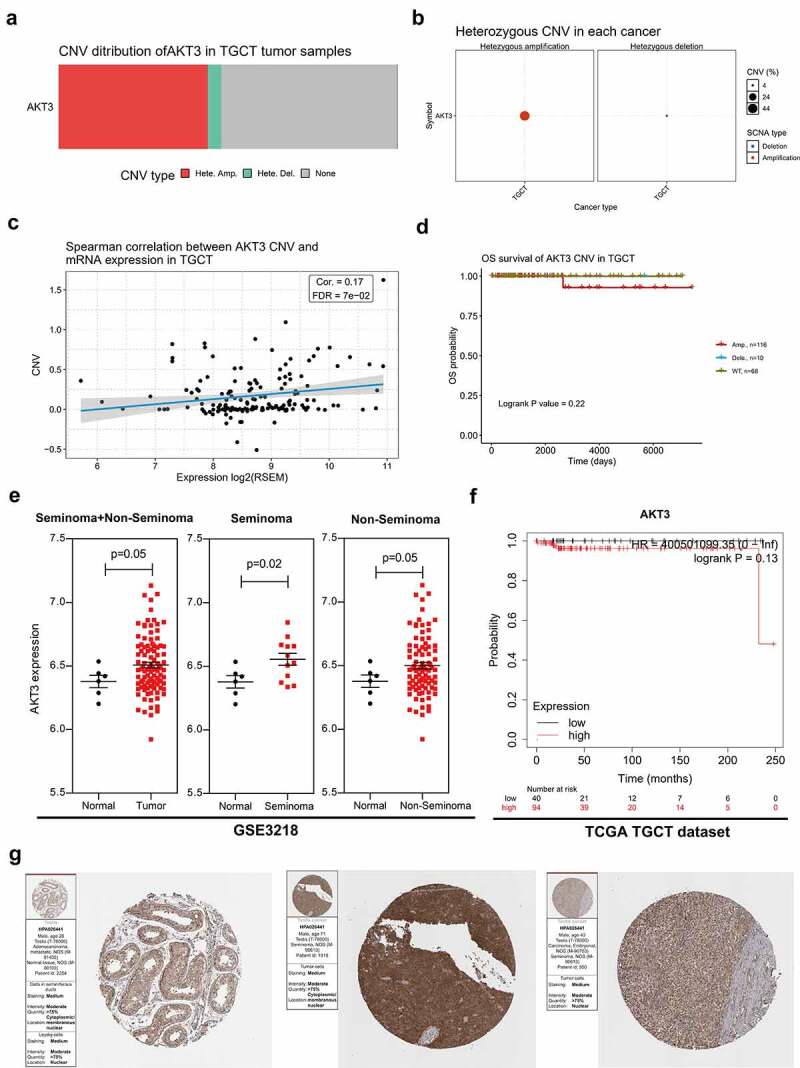
**AKT3 high copy number and expression correlates to TGCT patients prognosis**. (a) 100% Stacked Column Chart showing CNV of the AKT3 gene in the TCGA-TGCT cohort. (b) Bubble Chart showing CNV type of the AKT3 gene in the TCGA-TGCT cohort. (c) Correlation between AKT3 CNV and mRNA expression in the TCGA-TGCT cohort. (d) Correlation between AKT3 CNV and the survival outcome of patients in the TCGA-TGCT cohort. (e) AKT3 differential expression in tumor versus normal tissues from GSE3218. (f) Correlation between AKT3 mRNA expression and the survival outcome of patients from the TCGA-TGCT cohort. (g) AKT3 protein levels in normal testis and TGCT

In the TCGA-TGCT cohort, CNVs were mainly manifested by heterozygous amplification, along with heterozygous deletion in a very small number of samples (Figure 1a-b). Additionally, a positive correlation was detected between AKT3 mRNA expression and CNVs (Figure 1c). Patients with heterozygous amplification tended to have a worse outcome than those with copy number deletion and wild-type AKT3 (Figure 1d). Also, we found that AKT3 expression levels in tumor tissues were higher than in normal ones (Figure 1e). Based on TCGA-TGCT data, higher AKT3 mRNA expressions (cut off value = 487) tended to correlate to a poorer overall survival outcome (figure 1f, Table 2). However, no statistical difference was detected due to the small sample size or the higher survival rate of patients. Finally, we found that AKT3 proteins increased in TGCT (Figure 1g).

**Table 2.** The clinical follow-up data in TCGA-TGCT dataset

sample	OS	_PATIENT	OS.time
TCGA-4 K-AA1I-01A	0	TCGA-4 K-AA1I	3
TCGA-XE-AAOL-01A	0	TCGA-XE-AAOL	13
TCGA-XE-AANR-01A	0	TCGA-XE-AANR	14
TCGA-YU-A90Y-01A	1	TCGA-YU-A90Y	17
TCGA-SB-A76 C-01A	0	TCGA-SB-A76C	42
TCGA-W4-A7U3-01A	0	TCGA-W4-A7U3	149
TCGA-XE-A8H1-01A	0	TCGA-XE-A8H1	209
TCGA-XE-AAO6-01A	0	TCGA-XE-AAO6	240
TCGA-XE-AAOC-01A	0	TCGA-XE-AAOC	270
TCGA-S6-A8JY-01A	0	TCGA-S6-A8JY	278
TCGA-XY-A9T9-01A	0	TCGA-XY-A9T9	281
TCGA-SN-A84W-01A	0	TCGA-SN-A84W	293
TCGA-SN-A84X-01A	0	TCGA-SN-A84X	312
TCGA-XE-AAO4-01A	0	TCGA-XE-AAO4	367
TCGA-SB-A6J6-01A	0	TCGA-SB-A6J6	413
TCGA-2X-A9D5-01A	0	TCGA-2X-A9D5	435
TCGA-4 K-AA1G-01A	0	TCGA-4 K-AA1G	436
TCGA-XY-A89B-01A	0	TCGA-XY-A89B	471
TCGA-4 K-AAAL-01A	0	TCGA-4 K-AAAL	483
TCGA-SN-A6IS-01A	0	TCGA-SN-A6IS	496
TCGA-2 G-AAFM-01A	0	TCGA-2 G-AAFM	503
TCGA-XE-AAOB-01A	1	TCGA-XE-AAOB	513
TCGA-4 K-AA1H-01A	0	TCGA-4 K-AA1H	518
TCGA-SN-A84Y-01A	0	TCGA-SN-A84Y	524
TCGA-YU-A94I-01A	0	TCGA-YU-A94I	536
TCGA-SO-A8JP-01A	0	TCGA-SO-A8JP	540
TCGA-2 G-AAHT-01A	0	TCGA-2 G-AAHT	542
TCGA-S6-A8JX-01A	0	TCGA-S6-A8JX	552
TCGA-XE-A8H4-01A	0	TCGA-XE-A8H4	559
TCGA-2X-A9D6-01A	0	TCGA-2X-A9D6	607
TCGA-2 G-AAEW-01A	1	TCGA-2 G-AAEW	618
TCGA-ZM-AA0N-01A	0	TCGA-ZM-AA0N	634
TCGA-XY-A8S2-01A	0	TCGA-XY-A8S2	672
TCGA-2 G-AAFI-01A	0	TCGA-2 G-AAFI	675
TCGA-ZM-AA0F-01A	0	TCGA-ZM-AA0F	681
TCGA-2 G-AAFO-01A	0	TCGA-2 G-AAFO	685
TCGA-S6-A8JW-01A	0	TCGA-S6-A8JW	698
TCGA-XE-A9SE-01A	0	TCGA-XE-A9SE	708
TCGA-VF-A8AE-01A	0	TCGA-VF-A8AE	727
TCGA-W4-A7U4-01A	0	TCGA-W4-A7U4	738
TCGA-2 G-AAFL-01A	0	TCGA-2 G-AAFL	750
TCGA-WZ-A7V3-01A	0	TCGA-WZ-A7V3	753
TCGA-VF-A8AB-01A	0	TCGA-VF-A8AB	760
TCGA-2 G-AAFN-01A	0	TCGA-2 G-AAFN	773
TCGA-WZ-A8D5-01A	0	TCGA-WZ-A8D5	774
TCGA-2 G-AAFJ-01A	0	TCGA-2 G-AAFJ	792
TCGA-ZM-AA0E-01A	0	TCGA-ZM-AA0E	811
TCGA-ZM-AA0B-01A	0	TCGA-ZM-AA0B	838
TCGA-XY-A8S3-01B	0	TCGA-XY-A8S3	843
TCGA-ZM-AA0D-01A	0	TCGA-ZM-AA0D	848
TCGA-X3-A8G4-01A	0	TCGA-X3-A8G4	856
TCGA-YU-AA61-01A	0	TCGA-YU-AA61	864
TCGA-YU-A912-01A	0	TCGA-YU-A912	866
TCGA-WZ-A7V4-01A	0	TCGA-WZ-A7V4	894
TCGA-YU-A90S-01A	0	TCGA-YU-A90S	971
TCGA-VF-A8AD-01A	0	TCGA-VF-A8AD	1006
TCGA-XE-AANI-01A	0	TCGA-XE-AANI	1041
TCGA-WZ-A7V5-01A	0	TCGA-WZ-A7V5	1061
TCGA-VF-A8AC-01A	0	TCGA-VF-A8AC	1083
TCGA-2 G-AAFY-01A	0	TCGA-2 G-AAFY	1099
TCGA-YU-A90W-01A	0	TCGA-YU-A90W	1113
TCGA-VF-A8A9-01A	0	TCGA-VF-A8A9	1119
TCGA-ZM-AA05-01A	0	TCGA-ZM-AA05	1126
TCGA-VF-A8A8-01A	0	TCGA-VF-A8A8	1131
TCGA-VF-A8AA-01A	0	TCGA-VF-A8AA	1146
TCGA-XE-A8H5-01A	0	TCGA-XE-A8H5	1217
TCGA-2 G-AAEX-01A	0	TCGA-2 G-AAEX	1259
TCGA-W4-A7U2-01A	0	TCGA-W4-A7U2	1263
TCGA-XE-AAOF-01A	0	TCGA-XE-AAOF	1268
TCGA-2 G-AAFZ-01A	0	TCGA-2 G-AAFZ	1353
TCGA-2 G-AAFH-01A	0	TCGA-2 G-AAFH	1375
TCGA-2 G-AAFV-01A	0	TCGA-2 G-AAFV	1389
TCGA-2 G-AAF1-01A	0	TCGA-2 G-AAF1	1403
TCGA-ZM-AA06-01A	0	TCGA-ZM-AA06	1498
TCGA-2 G-AAG0-01A	0	TCGA-2 G-AAG0	1529
TCGA-XE-AAOJ-01A	0	TCGA-XE-AAOJ	1550
TCGA-YU-AA4L-01A	0	TCGA-YU-AA4L	1573
TCGA-2 G-AAG3-01A	0	TCGA-2 G-AAG3	1593
TCGA-XE-AANV-01A	0	TCGA-XE-AANV	1701
TCGA-ZM-AA0H-01A	0	TCGA-ZM-AA0H	1736
TCGA-2 G-AAHG-01A	0	TCGA-2 G-AAHG	1819
TCGA-2 G-AAH3-01A	0	TCGA-2 G-AAH3	1822
TCGA-YU-A90Q-01A	0	TCGA-YU-A90Q	1964
TCGA-XE-AANJ-01A	0	TCGA-XE-AANJ	2007
TCGA-XE-AAOD-01A	0	TCGA-XE-AAOD	2058
TCGA-YU-A90P-01A	0	TCGA-YU-A90P	2069
TCGA-YU-A94D-01A	0	TCGA-YU-A94D	2167
TCGA-2 G-AAG6-01A	0	TCGA-2 G-AAG6	2231
TCGA-2 G-AAG9-01A	0	TCGA-2 G-AAG9	2234
TCGA-2 G-AAH8-01A	0	TCGA-2 G-AAH8	2248
TCGA-2 G-AAG8-01A	0	TCGA-2 G-AAG8	2289
TCGA-2 G-AAG5-01A	0	TCGA-2 G-AAG5	2299
TCGA-2 G-AAF4-01A	0	TCGA-2 G-AAF4	2315
TCGA-2 G-AAGA-01A	0	TCGA-2 G-AAGA	2439
TCGA-2 G-AAG7-01A	0	TCGA-2 G-AAG7	2475
TCGA-2 G-AAGE-01A	0	TCGA-2 G-AAGE	2478
TCGA-2 G-AAGC-01A	0	TCGA-2 G-AAGC	2585
TCGA-2 G-AAGG-01A	0	TCGA-2 G-AAGG	2611
TCGA-2 G-AALP-01A	0	TCGA-2 G-AALP	2645
TCGA-2 G-AAGF-01A	0	TCGA-2 G-AAGF	2734
TCGA-XE-AAO3-01A	0	TCGA-XE-AAO3	2857
TCGA-2 G-AAGJ-01A	0	TCGA-2 G-AAGJ	2938
TCGA-2 G-AAGI-01A	0	TCGA-2 G-AAGI	3137
TCGA-2 G-AAGI-05A	0	TCGA-2 G-AAGI	3137
TCGA-2 G-AAHA-01A	0	TCGA-2 G-AAHA	3349
TCGA-2 G-AAF6-01A	0	TCGA-2 G-AAF6	3491
TCGA-2 G-AAGK-01A	0	TCGA-2 G-AAGK	3621
TCGA-2 G-AAGN-01A	0	TCGA-2 G-AAGN	3656
TCGA-2 G-AAGM-01A	0	TCGA-2 G-AAGM	3891
TCGA-2 G-AAF8-01A	0	TCGA-2 G-AAF8	3911
TCGA-2 G-AAL7-01A	0	TCGA-2 G-AAL7	3991
TCGA-2 G-AAGP-01A	0	TCGA-2 G-AAGP	4018
TCGA-2 G-AAGO-01A	0	TCGA-2 G-AAGO	4026
TCGA-2 G-AAGT-01A	0	TCGA-2 G-AAGT	4406
TCGA-2 G-AAGV-01A	0	TCGA-2 G-AAGV	4527
TCGA-2 G-AAFE-01A	0	TCGA-2 G-AAFE	4702
TCGA-2 G-AAGS-01A	0	TCGA-2 G-AAGS	4816
TCGA-2 G-AAGW-01A	0	TCGA-2 G-AAGW	4899
TCGA-2 G-AAGZ-01A	0	TCGA-2 G-AAGZ	5233
TCGA-2 G-AAGY-01A	0	TCGA-2 G-AAGY	5329
TCGA-2 G-AAGY-05A	0	TCGA-2 G-AAGY	5329
TCGA-2 G-AAH0-01A	0	TCGA-2 G-AAH0	5477
TCGA-2 G-AAGX-01A	0	TCGA-2 G-AAGX	5514
TCGA-2 G-AAHC-01A	0	TCGA-2 G-AAHC	5539
TCGA-2 G-AAHP-05A	0	TCGA-2 G-AAHP	5667
TCGA-2 G-AAHP-01A	0	TCGA-2 G-AAHP	5667
TCGA-2 G-AAH2-01A	0	TCGA-2 G-AAH2	6060
TCGA-2 G-AAFG-01A	0	TCGA-2 G-AAFG	6183
TCGA-2 G-AAFG-05A	0	TCGA-2 G-AAFG	6183
TCGA-2 G-AAH4-01A	0	TCGA-2 G-AAH4	6210
TCGA-2 G-AAKD-01A	0	TCGA-2 G-AAKD	6340
TCGA-2 G-AAHN-01A	0	TCGA-2 G-AAHN	6445
TCGA-2 G-AAKG-01A	0	TCGA-2 G-AAKG	6598
TCGA-2 G-AAKG-05A	0	TCGA-2 G-AAKG	6598
TCGA-2 G-AAKH-01A	0	TCGA-2 G-AAKH	6862
TCGA-2 G-AAKM-01A	1	TCGA-2 G-AAKM	6972
TCGA-2 G-AAKL-01A	0	TCGA-2 G-AAKL	7023
TCGA-2 G-AAHL-01A	0	TCGA-2 G-AAHL	7081
TCGA-2 G-AAL5-01A	0	TCGA-2 G-AAL5	7437

### AKT3 mutations are correlated to moderate survival outcomes in TGCT patients

**Figure 2.**
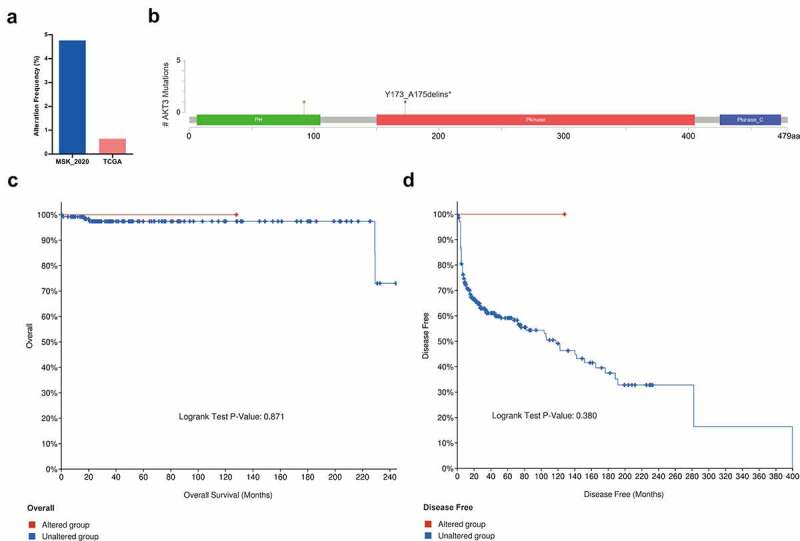
**AKT3 mutation is associated with survival outcomes of TGCT patients**. (a) Mutation frequency of AKT3 in different TGCT cohorts in the cBioPortal database. (b) The position of the AKT3 mutation site on the AKT3 protein. (c) Association of AKT3 mutation with overall survival. (d) Association of AKT3 mutation with disease-free survival

Mutation analysis showed a low AKT3 mutation frequency in TGCT (Figure 2a). Mutations were mainly located in the PH and Pkinase functional domains (Figure 2b). Furthermore, we demonstrated that patients with AKT3 mutations had better overall and recurrence-free survivals (Figure 2c-d).

### AKT3 promotes NCCIT cells’ proliferation and colony formation

**Figure 3.**
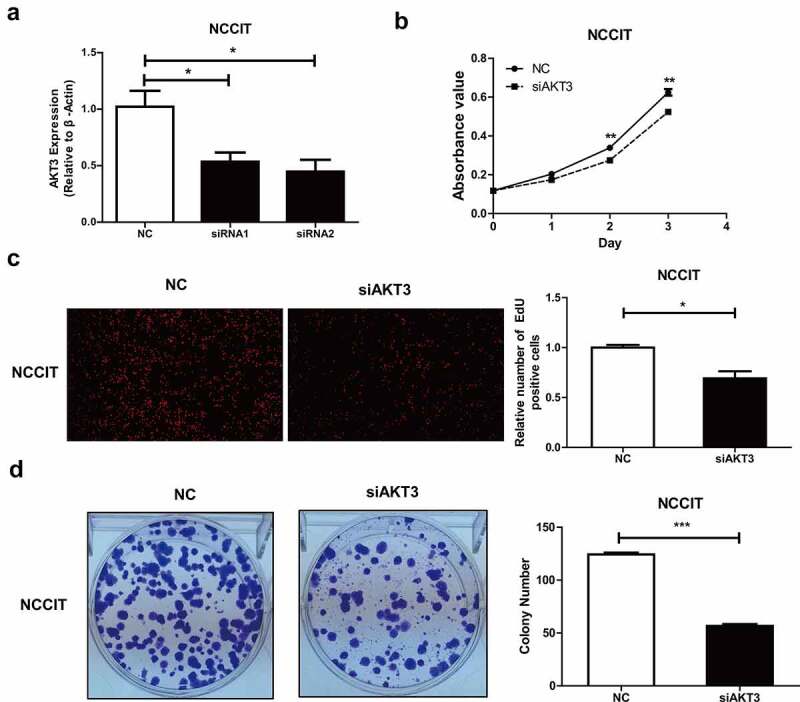
**Effect of AKT3 silencing on NCCIT cells biological functions**. (a) The silencing effect of AKT3 siRNA by qRT-PCR. (b) MTT assay showing cell proliferation upon AKT3 silencing. (c) EdU assay showing DNA synthesis ability of AKT3-silenced NCCIT cells. (d) Colony formation assay showing cell colony formation ability after AKT3 silencing. *p< 0.05, **p< 0.01, ***p< 0.001

Further, we conducted *in vitro* experiments to evaluate AKT3’s biological functions. First, NCCIT cells were treated by AKT3-siRNA to create an AKT3-silencing cell model. Two siRNAs (siRNA1, siRNA2) were used. The siRNA2 had a better silencing effect and was selected for subsequent experiments (Figure 3a). MTT results revealed reduced proliferative ability of cells upon AKT3 silencing (Figure 3b). Similarly, the DNA synthesis and colony formation ability of NCCIT cells significantly decreased when AKT3 expression was silenced (Figure 3c-d).

### AKT3 can regulate multiple signaling pathways in TGCT

**Figure 4.**
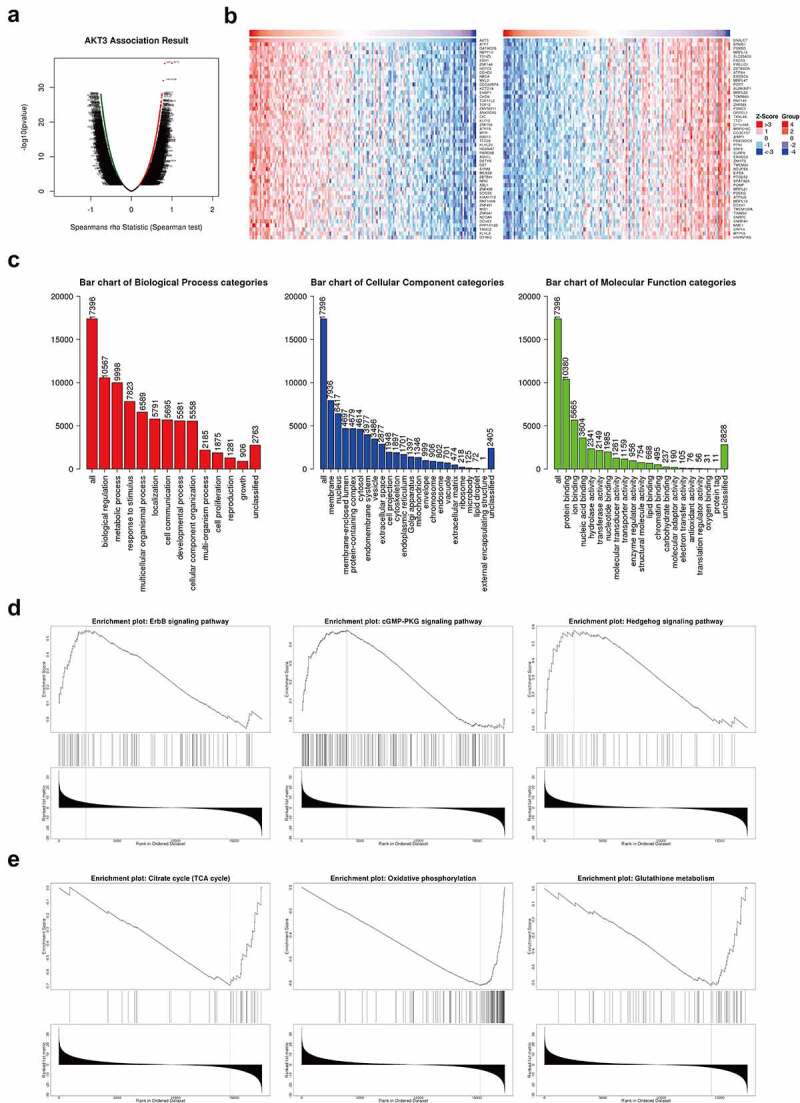
**Enrichment analysis of AKT3 co-expressed genes**. (a) Volcano map of genes significantly associated with AKT3. (b) Heat Map showing genes that are significantly positively and negatively related to AKT3. (c) Most enriched GO terms from Biological Process, Cellular Component, and Molecular Function. D, (e) Most enriched KEGG pathways

We obtained 5,036 positively and 3,849 negatively correlated genes with AKT3 expression (Figure 4a), and the top 50 are displayed on the heat map (Figure 4b, Table 3). These genes were significantly enriched for cancer-promoting terms, such as cell proliferation, growth, cytoskeleton, nucleic acid binding, enzyme regulatory activity (Figure 4c). Moreover, KEGG analysis indicated significant enrichment in ErbB, cGMP-PKG, and Hedgehog signaling pathways. The TCA cycle, oxidative phosphorylation, and glutathione metabolism signaling pathways were also significantly enriched (Figure 4d/E).

**Table 3.** Each 50 gene which showed positive and negative correlation with AKT3 expression based on TCGA-TGCT dataset

Query	Statistic	P-value	FDR (BH)	Event_SD	Event_TD
AKT3	1	1E-37	1E-33	150	150
ATF7	0.81977	1.18E-37	1.18E-33	150	150
GATAD2B	0.786044	1.01E-32	6.71E-29	150	150
NBPF10	0.755203	6.13E-29	3.07E-25	150	150
TSHZ3	0.751794	1.48E-28	3.4E-25	150	150
SSH1	0.751679	1.53E-28	3.4E-25	150	150
ZNF148	0.749226	2.86E-28	5.21E-25	150	150
HCFC2	0.744733	8.86E-28	1.27E-24	150	150
DDHD2	0.73537	8.66E-27	7.87E-24	150	150
NBEA	0.734478	1.07E-26	9.31E-24	150	150
MKL2	0.73403	1.19E-26	9.92E-24	150	150
CDC42BPA	0.732506	1.71E-26	1.36E-23	150	150
KCTD18	0.731311	2.26E-26	1.69E-23	150	150
EHBP1	0.725181	9.3E-26	5.43E-23	150	150
CHD9	0.72372	1.3E-25	7.2E-23	150	150
TCP11 L2	0.722713	1.63E-25	8.79E-23	150	150
TCF12	0.722155	1.84E-25	9.7E-23	150	150
ZMYND11	0.72002	2.97E-25	1.45E-22	150	150
ANKRD52	0.719055	3.68E-25	1.71E-22	150	150
CIC	0.718193	4.46E-25	2.03E-22	150	150
KLF12	0.716434	6.56E-25	2.79E-22	150	150
ZNF704	0.711462	1.93E-24	7.57E-22	150	150
ATP7A	0.710526	2.36E-24	9.07E-22	150	150
MTR	0.707739	4.26E-24	1.55E-21	150	150
BBS10	0.70709	4.89E-24	1.72E-21	150	150
TTC28	0.707071	4.91E-24	1.72E-21	150	150
KLHL20	0.706738	5.26E-24	1.82E-21	150	150
HGSNAT	0.704776	7.94E-24	2.65E-21	150	150
PARD3B	0.702883	1.18E-23	3.8E-21	150	148
ASH1L	0.702325	1.32E-23	4.13E-21	150	150
DSTYK	0.701939	1.43E-23	4.4E-21	150	150
DST	0.700089	2.09E-23	6.24E-21	150	150
SYNM	0.698416	2.94E-23	8.29E-21	150	150
MEX3B	0.697255	3.72E-23	9.79E-21	150	150
ZBTB41	0.697182	3.78E-23	9.81E-21	150	150
NFIC	0.696779	4.1E-23	1.05E-20	150	150
ABL1	0.696285	4.53E-23	1.15E-20	150	150
ZNF436	0.695884	4.91E-23	1.21E-20	150	150
SOCS5	0.695716	5.07E-23	1.24E-20	150	150
KIAA1715	0.695128	5.71E-23	1.31E-20	150	150
RNF144A	0.694047	7.09E-23	1.59E-20	150	150
ZNF491	0.69395	7.23E-23	1.61E-20	150	150
MIB1	0.693735	7.54E-23	1.66E-20	150	150
ZNF641	0.692145	1.03E-22	2.23E-20	150	150
NCOA6	0.691499	1.18E-22	2.48E-20	150	150
DCHS1	0.69041	1.46E-22	2.97E-20	150	150
PPP1R12B	0.68942	1.77E-22	3.58E-20	150	150
TANC2	0.687768	2.44E-22	4.76E-20	150	150
KLHL8	0.687539	2.55E-22	4.91E-20	150	150
DYRK2	0.687171	2.74E-22	5.2E-20	150	150
DNAJC7	−0.75356	9.4E-29	3.4E-25	150	150
BRMS1	−0.75306	1.07E-28	3.4E-25	150	150
PSMB3	−0.7523	1.3E-28	3.4E-25	150	150
MRPL16	−0.75011	2.28E-28	4.57E-25	150	150
SLC25A39	−0.74815	3.76E-28	6.26E-25	150	150
FADS3	−0.74499	8.32E-28	1.27E-24	150	150
PRELID1	−0.74396	1.07E-27	1.34E-24	150	150
ZBTB8OS	−0.74396	1.07E-27	1.34E-24	150	150
ATP5H	−0.74083	2.32E-27	2.73E-24	150	150
EXOSC8	−0.74033	2.62E-27	2.91E-24	150	150
MRPL47	−0.74003	2.82E-27	2.97E-24	150	150
POP7	−0.73611	7.26E-27	7.26E-24	150	150
AURKAIP1	−0.7354	8.6E-27	7.87E-24	150	150
MRPL22	−0.73126	2.29E-26	1.69E-23	150	150
TOMM40	−0.72945	3.48E-26	2.49E-23	150	150
RNF181	−0.72911	3.77E-26	2.6E-23	150	150
ZNF593	−0.72818	4.67E-26	3.12E-23	150	150
PSMC5	−0.72737	5.63E-26	3.63E-23	150	150
GRPEL1	−0.72615	7.45E-26	4.66E-23	150	150
TXNL4A	−0.72539	8.87E-26	5.38E-23	150	150
TTC1	−0.72508	9.5E-26	5.43E-23	150	150
C11orf48	−0.72131	2.23E-25	1.14E-22	150	150
MRPS18C	−0.72118	2.3E-25	1.15E-22	150	150
CCDC137	−0.71958	3.27E-25	1.56E-22	150	150
AIMP1	−0.71734	5.37E-25	2.39E-22	150	150
FBXO22OS	−0.71696	5.85E-25	2.54E-22	150	150
PFN1	−0.71552	8.01E-25	3.34E-22	150	150
SNF8	−0.71381	1.16E-24	4.74E-22	150	150
SURF6	−0.71293	1.41E-24	5.62E-22	150	150
EXOSC9	−0.70936	3.02E-24	1.14E-21	150	150
ZNHIT3	−0.70803	4.01E-24	1.48E-21	150	150
TMEM93	−0.70489	7.76E-24	2.63E-21	150	150
NDUFS6	−0.7046	8.23E-24	2.7E-21	150	150
EIF5A	−0.70235	1.31E-23	4.13E-21	150	150
PTGES2	−0.70055	1.9E-23	5.76E-21	150	150
SPATA24	−0.69945	2.38E-23	7.01E-21	150	150
POMP	−0.69921	2.5E-23	7.26E-21	150	150
MRPL21	−0.6991	2.56E-23	7.3E-21	150	150
PDE6G	−0.69826	3.03E-23	8.43E-21	150	150
ATP5J2	−0.69774	3.37E-23	9.24E-21	150	150
MRPL12	−0.69764	3.44E-23	9.3E-21	150	150
DDX41	−0.69732	3.67E-23	9.79E-21	150	150
TMEM126A	−0.69617	4.64E-23	1.16E-20	150	150
TIMM50	−0.69558	5.21E-23	1.26E-20	150	150
SNRPC	−0.69549	5.31E-23	1.26E-20	150	150
SNRPA1	−0.6952	5.63E-23	1.31E-20	150	150
NME1	−0.69519	5.64E-23	1.31E-20	150	150
SRP19	−0.69508	5.77E-23	1.31E-20	150	150
MTP18	−0.69318	8.42E-23	1.83E-20	150	150
HNRNPAB	−0.69162	1.15E-22	2.44E-20	150	150

### AKT3 mRNA expression might be regulated by DNA methylation

**Figure 5.**
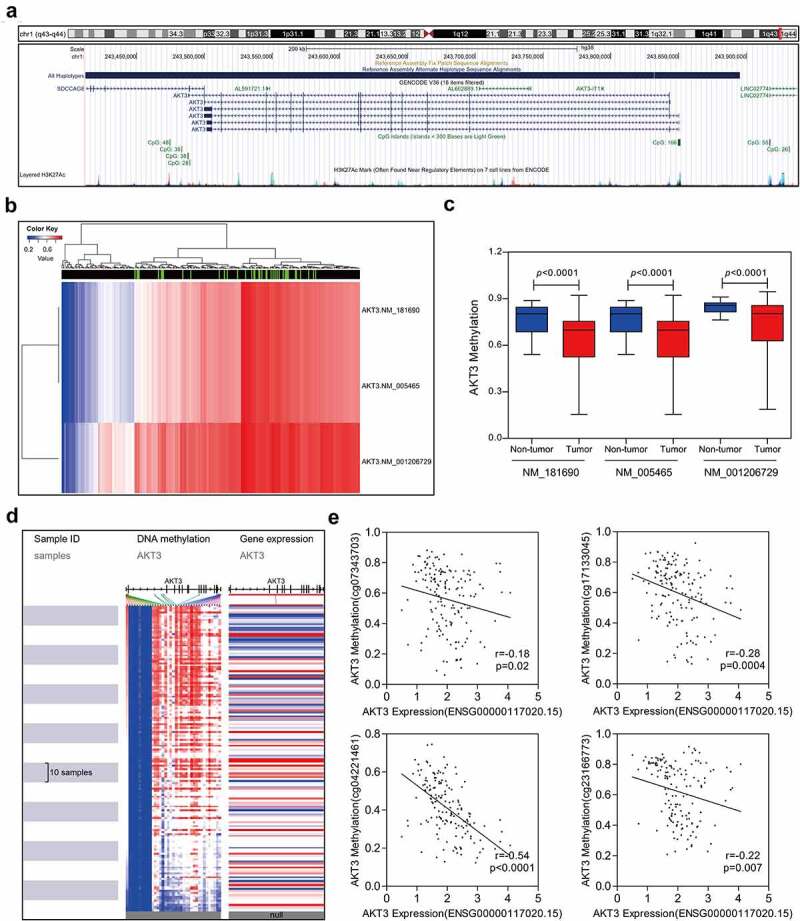
**Correlation between AKT3 DNA methylation and mRNA expression level**. (a) Distribution of AKT3 CpG islands. (b) Heatmap showing DNA methylation levels in different AKT3 subtypes. (c) The methylation level of AKT3 significantly reduced in TGCT samples. (d) Heatmap of AKT3 DNA methylation and expression levels. (e) Correlation between signal values of different AKT3 methylation probes and mRNA expression

DNA methylation can participate in the regulation of various genes. Multiple CpG islands were noted around the AKT3 promoter region (Figure 5a). Then, the methylation level of AKT3 was retrieved from the TGCT methylation data. We found that all AKT3 transcripts were methylated to varying degrees (Figure 5b), and the level was significantly lower in TGCT tumor tissues compared to controls (Figure 5c). Moreover, the methylation signal intensity on CpG islands was negatively correlated to AKT3 mRNA expression levels (Figure 5d). Finally, four methylation signal probe datasets were randomly selected and demonstrated a clear negative correlation between AKT3 methylation and mRNA expression levels (Figure 5e).

### AKT3 expression correlates to TGCT immune infiltration and drug sensitivity

**Figure 6.**
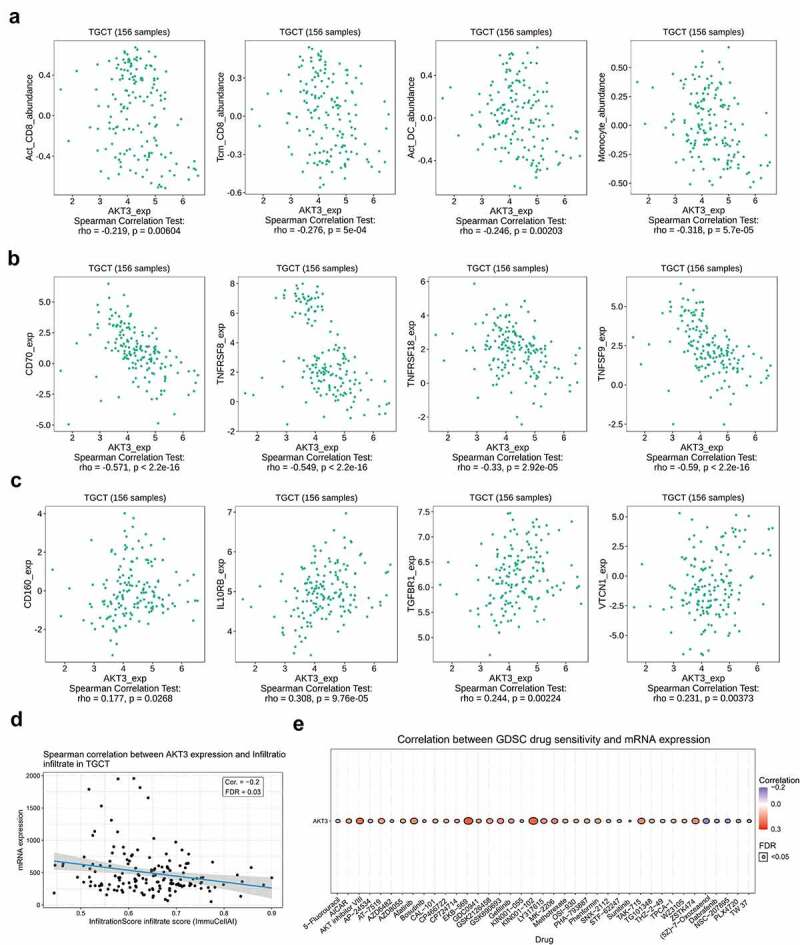
**AKT3 is associated with tumor immune infiltration and drug sensitivity**. (a) Correlation with different types of immune infiltrates. (b) Correlation with immune-activating molecules. (c) Correlation with immunosuppressive molecules. (d) Correlation with the TGCT overall immune infiltration. (e) Correlation with the sensitivity to multiple drugs

Many studies have shown that tumor immune infiltrates are involved in the occurrence and development of tumors and can be potential prognostic markers for patients’ survival outcomes. Here, we demonstrated a significantly negative correlation of AKT3 expression with the abundance of tumor-infiltrating cells, including activated CD8 + T cells, CD8+ memory T cells, activated dendritic cells, and monocytes (Figure 6a). Besides, the AKT3 expression was related to many immune-related molecules. We found that the AKT3 expression significantly negatively correlated to immune-activating molecules, including CD70, TNFRSF8, TNFRSF18, TNFSF9 (Figure 6b); while positively correlated to immunosuppressive molecules, including CD160, IL10RB, TGFBR1, and VTCN1 (Figure 6c). Additionally, we detected a significantly negative correlation between AKT3 expression and immune infiltration scores (Figure 6d). The drug sensitivity analysis revealed a positive correlation between the AKT3 expression and the sensitivity to different drugs (Figure 6e). The AKT3 expression was significantly positively correlated with the sensitivity to AKT inhibitors VIII, MK-2206, and GSK690693. Also, its expression level was related to the sensitivity to the CDK inhibitor AT-7519. These data suggest that these inhibitors can be used for AKT3 *in vivo* experiments, especially AKT inhibitors VIII, MK-2206, and GSK690693.

## Discussion

Multiple studies have demonstrated that AKT3 is involved in almost all processes during tumor initiation and progression, including proliferation, migration, invasion, and drug resistance. For example, a prostate cancer study found that AKT3 overexpression could lead to increased resistance of differentiated neuroendocrine tumor cells to androgen therapy [23]. In interstitial colorectal cancer, AKT3 played a promotive role in tumor cell growth and was potentially correlated to the malignant epithelial-mesenchymal transition (EMT) [24]. On the other hand, different AKT3 functions have been reported. For example, a team from the Universität Hamburg, Germany, found that AKT3 silencing contributed to increased invasion and migration of breast cancer cells by activating HER2 and DDR signals [25]. The aforementioned studies reflect the complexity and diversity of AKT3 molecular functions. Thus, more in-depth studies should be conducted. In the current study, we demonstrated for the first time the promotive role of AKT3 expression in the proliferation and colony formation of NSE cells, indicative of the potential cancer-promoting role of AKT3 in TGCT, especially NSE. A further in-depth study on the underlying mechanism might help AKT3 become a molecular target for NSE treatments.

Also, AKT3 co-expressed genes were identified and annotated by GO analysis. The results revealed significant enrichment of biological processes related to cell proliferation, indicating the role of AKT3 in the regulation of this process. Additionally, KEGG pathway enrichment analysis showed that AKT3 positive-related genes were significantly enriched in cancer-promoting signaling pathways such as ErbB and Hedgehog, suggesting that, in TGCT, AKT3 is more likely to promote tumor progression. Besides, AKT3 negative-related genes were found highly activated in metabolism-related signaling pathways such as the TCA cycle, oxidative phosphorylation, and glutathione metabolism, which suggested the involvement of AKT3 in the energy metabolism and oxidative stress of tumor cells. However, the mechanisms underlying the AKT3 participation in these signaling pathways remain unclear.

DNA methylation is essential for gene transcriptional regulation and generally serves as a determinant of transcriptional activity [26,27]. Previous studies revealed that DNA methylation was closely related to cisplatin resistance in TCGT patients [28]. Also, the high expression of DNA methyltransferase 3B was related to the sensitivity of TGCT to 5-aza-deoxycytidine [29]. In the present study, data from multiple databases showed significantly hypomethylated AKT3 promoters in TGCT, which was negatively correlated with AKT3 mRNA expression levels. This suggested that AKT3 upregulation is highly likely a result of AKT3 promoter demethylation. Therefore, drugs targeting DNA methylation can be regarded as a new treatment strategy for TGCT.

The tumor immune microenvironment is closely related to tumor progression and treatment. Studies revealed that the abundance of immune infiltrates is significantly related to the outcome of TGCT patients. Also, low infiltration abundances of CD4 + T and CD8 + T cells generally indicate a higher recurrence rate [30–32]. Also, it was reported that AKT3, despite its regulatory role in proliferation and apoptosis, was associated with the infiltration of various immune cells in tumor tissues, including T cells and macrophages [33]. TLR2 is reported to be positively correlated with M0 macrophage infiltration and negatively correlated with naive B and follicular helper T cells in TGCT [34]. In the current study, we found a new marker of TGCT immune infiltration. Based on the TISIDB database, we found a negative correlation between AKT3 expression and the abundance of immune infiltrates, including CD8 + T cells. Additionally, immune-related genes have been previously related to the prognosis of TGCT patients [35]. Here, we found that AKT3 expression was negatively correlated with four immune-activating genes, while positively correlated with four immunosuppressive genes. Therefore, AKT3 might play an important role in TGCT anti-tumor immunity.

### Limitations

Our study also has some limitations. For example, our results were obtained from *in vitro* studies and *in vivo* experiments are required in the future. Also, the specific molecular mechanism by which AKT3 promotes NCCIT cell proliferation has not been clarified, and further molecular experiments are still needed.

### Conclusions & future perspectives

Overall, we showed an increased AKT3 expression in TGCT patients and identified its associations with poor survival outcomes and immune infiltration. AKT3 could also promote the proliferation, DNA synthesis and colony formation of NES cells *in vitro*. AKT3 might be a potential therapeutic target and a novel molecular marker of TGCT.

## Data Availability

The data supporting the findings reported in this study are available from the corresponding author upon reasonable request.

## List of abbreviations

TGCT: testicular germ cell tumors; SE: seminomas; NSE: non-seminomas; RPMI: Roswell Park Memorial Institute; FBS: fetal bovine serum; siRNA: small interfering RNA; qRT-PCR: quantitative real-time polymerase chain reaction; GEO: gene expression omnibus; HPA: Human Protein Atlas; TCGA: The Cancer Genome Atlas; CNVs: copy number variations; ANOVA: analysis of variance; GSEA: Gene Set Enrichment Analysis; cDNA: complementary DNA; GSCA: Gene Set Cancer Analysis; EMT: epithelial-mesenchymal transition.
